# The secondary spiral lamina and its relevance in cochlear implant surgery

**DOI:** 10.1080/03009734.2018.1443983

**Published:** 2018-03-14

**Authors:** Sumit Agrawal, Nadine Schart-Morén, Wei Liu, Hanif M. Ladak, Helge Rask-Andersen, Hao Li

**Affiliations:** a Department of Otolaryngology-Head and Neck Surgery, Western University, London, ON, Canada; b Department of Surgical Sciences, Section of Otolaryngology, Department of Otolaryngology, Uppsala University Hospital, Uppsala, Sweden; c Department of Medical Biophysics and Department of Electrical and Computer Engineering, Western University, London, ON, Canada

**Keywords:** Basilar membrane, cochlea, human, secondary spiral lamina, synchrotron-phase contrast imaging

## Abstract

**Objective:**

We used synchrotron radiation phase contrast imaging (SR-PCI) to study the 3D microanatomy of the basilar membrane (BM) and its attachment to the spiral ligament (SL) (with a conceivable secondary spiral lamina [SSL] or secondary spiral plate) at the round window membrane (RWM) in the human cochlea. The conception of this complex anatomy may be essential for accomplishing structural preservation at cochlear implant surgery.

**Material and methods:**

Sixteen freshly fixed human temporal bones were used to reproduce the BM, SL, primary and secondary osseous spiral laminae (OSL), and RWM using volume-rendering software. Confocal microscopy immunohistochemistry (IHC) was performed to analyze the molecular constituents.

**Results:**

SR-PCI reproduced the soft tissues including the RWM, Reissner’s membrane (RM), and the BM attachment to the lateral wall (LW) in three dimensions. A variable SR-PCI contrast enhancement was recognized in the caudal part of the SL facing the scala tympani (ST). It seemed to represent a SSL allied to the basilar crest (BC). The SSL extended along the postero-superior margin of the round window (RW) and immunohistochemically expressed type II collagen.

**Conclusions:**

Unlike in several mammalian species, the human SSL is restricted to the most basal portion of the cochlea around the RW. It anchors the BM and may influence its hydro-mechanical properties. It could also help to shield the BM from the RW. The microanatomy should be considered at cochlear implant surgery.

## Introduction

The osseous spiral lamina (OSL) and the basilar crest (BC) form a ‘hammock’ to support the basilar membrane (BM) and the organ of Corti in the human cochlea. In most mammals, a secondary spiral lamina (SSL) forms a ridge on the outer wall that projects inward from the bony tube toward the primary lamina, leaving a narrow cleft for the BM. In the bat, which can perceive intense high-frequency sounds, the SSL is wide and supports the BM fibers firmly to the lateral wall (LW) (1). In the mouse and guinea pig, it is also prominent (2); however, in man, a species less adapted to perceive high-frequency sounds, it varies and is limited to the lower part of the basal turn (3) around the posterior and superior margins of the round window (RW) (4). The human BM is structurally modified from the base to the apex (width, thickness, and fiber characteristics), but also laterally with a resistance to displacement 100 times greater in the base than in the apex (5,6). The SSL may aid its suspension at the most basal part of the cochlea to maintain some BM stiffness. Hearing preservation cochlear implantation motivates further analysis of the intricate microanatomy at the cochlear base. We performed synchrotron radiation phase contrast imaging (SR-PCI) of 16 freshly fixed human temporal bones. The 3D-rendering software algorithms and color separations in each section were used to reconstruct various soft tissue components in the basal part of the cochlea with particular focus on the RW and SSL. In addition, confocal fluorescent microscopy was utilized to analyze the molecular components.

## Methods

### SR-PCI

*Temporal bone preparation*. The technique used in the present investigation was recently described by Elfarnawany et al. (7) and Koch et al. (8,9). A total of 16 fresh-frozen, then fixed, adult cadaveric temporal bones were used in this study. All specimens were obtained with permission from the Body Bequeathal Program at Western University, London, Ontario, Canada, in accordance with the Anatomy Act of Ontario and Western University’s Committee for Cadaveric Use in Research. After thawing, a cylindrical cutter was used to core a sample (40 mm diameter and 60 mm length) of the middle ear from each temporal bone. The samples were fixed in a 3.7% formaldehyde and 1% glutaraldehyde (4F-1G) buffer bath for 5 days. The samples were rinsed twice and dehydrated using an ethanol series (50%, 60%, 70%, 80%, 90%, 95%, and 100%). No additional processing (i.e. staining, sectioning, or decalcification) was performed on the samples. Sample fixation eliminated the risk of degradation during the 2-month time difference between imaging sessions and scanning. Samples were transferred to the imaging facilities in motion-proof containers to avoid the risk of damage during shipping.

*SR-PCI imaging*. The phase contrast imaging (PCI) technique used was in-line PCI, which has a setup similar to conventional radiography. It consists of an X-ray source, a sample, and a detector with no other optical elements. The detector is placed at a distance from the sample that allows the phase-shifted beam to interfere with the original beam and produce measurable fringes. The fringes correspond to surfaces and structural boundaries of the sample (edge enhancement) compared with a conventional radiogram. To obtain SR-PCI images, each sample was scanned using the Bio-Medical Imaging and Therapy (BMIT) 05ID-2 beamline at Canadian Light Source Inc. (CLSI) in Saskatoon, SK, Canada. It provides a synchrotron radiation (SR) beam produced by a superconducting wiggler source (10). The beam is filtered using a monochromator and yields an energy bandwidth of ΔE/E = 10^−3^ over an energy range of 25–150 keV (7). The imaging setup installed at the beamline length of 55 m from the source consists of a sample stage and a charge-coupled device-based detector system that are both placed on a vibration isolation table. The distance between the sample and detector was 2 m, and the photon energy was 47 keV. Motorized alignment stages were used to align the sample and detector for high-resolution tomography. The detector, an AA-60 beam monitor coupled with a C9300-124 camera (Hamamatsu Photonics, Shizuoka, Japan), has a 12-bit resolution and an effective pixel size of 9 × 9 mm^2^. The imaging field of view was set to 4000 × 950 pixels corresponding to 36.0 × 8.6 mm, and 3000 projections over 180 rotations were acquired per view. The 3D image volume had an isotropic voxel size of 9 mm. The acquisition time to capture all projections per view was ∼30 min. While computed tomography (CT) imaging is absorption-contrast based, PCI can potentially be combined with SRCT (SR-PCI, henceforth) to improve soft-tissue contrast while maintaining accurate visualization of bone. Conventional absorption-contrast based CT depends on the attenuation of X-rays, whereas in PCI the phase shift caused by the sample is transformed into detectable variations in X-ray intensity. PCI can provide edge enhancement by emphasizing the contrast between the boundaries of different structures in the image. The results demonstrate that SR-PCI can be used to simultaneously visualize both bone and soft tissues.

Anatomical structures of one cochlea were traced and color-labeled on serial sections (approx. 1,400) in three dimensions for one specimen (1552R) for 3D reconstruction. The data were fed into the software program 3D Slicer (www.slicer.org), and models were made using threshold paint tool in the editor module (11). A detailed comprehension of the soft tissue relationship of the basal part of the cochlea could be obtained rather than delineating them on a reconstructed 3D image. The relationship among the BM, the spiral ligament (SL), and the RW could be analyzed in 13 temporal bone specimens.

*Immunohistochemistry (IHC)*. This study on human materials was approved by the local ethics committee (no. 99398, 22/9 1999, cont., 2003 and Dnr. 2013/190), and patient consent was obtained. The studies adhered to the rules of the Declaration of Helsinki. The study used archival sections from prior studies, and the materials and methods were described there (12). Briefly, cochleae were dissected out as a whole piece during petro-clival meningioma surgery and immediately placed in a 4% paraformaldehyde solution diluted with phosphate buffered saline (PBS). After fixation, bones were placed in a 10% ethylenediaminetetraacetic acid (EDTA) solution for decalcification. The sections were embedded in Tissue-Tek OCT compound (Sakura Finetek, Zoeterwoude, The Netherlands), rapidly frozen, and then sectioned at 8–10 μm using a Leica cryostat microtome. The frozen sections were collected onto gelatin/chrome-alum-coated slides and stored below –70 °C before IHC. The antibody against laminin β2 was a rat monoclonal antibody at a dilution of 1:100 (05-206; Millipore, Billerica, MA, USA). It recognizes and is specific for the β2 chain laminin. The type IV collagen antibody was a goat polyclonal antibody at a dilution of 1:10 (AB769; Millipore). The antibody against neuron-specific class III beta-tubulin (Tuj1) was a polyclonal antibody at a dilution of 1:200 (04-1049; Millipore). Another tubulin antibody was a murine monoclonal antibody at a dilution of 1:200 (MAB1637; Millipore). Antibody combinations, characteristics, and sources are summarized in Table 1, and additional information can be found in the Discussion section. Elastin antibody was a murine monoclonal Ab (MAB2503; Millipore). IHC procedures on cochlear sections were described in previous publications (13,14). Briefly, incubation of sections on slides with a solution of the antibodies was carried out in a humid atmosphere at 4 °C for 20 h. After rinsing with PBS, the sections were incubated with secondary antibodies conjugated to Alexa Fluor 488 and 555 (Molecular Probes, Carlsbad, CA, USA), counter-stained with the nuclear stain 4’,6-diamidino-2-phenylindole dihydrochloride (DAPI) for 5 min, rinsed with PBS, and mounted with Vecta Shield mounting medium (Vector Laboratories, Burlingame, CA, USA). The sections used for antibody control were incubated with 2% bovine serum albumin (BSA) omitting the primary antibodies. As a result of the control experiment, there was no visible staining in any structure of the cochlea. Stained sections were investigated with an inverted fluorescence microscope (Nikon TE2000; Nikon Co., Tokyo, Japan) equipped with a spot digital camera with three filters (for emission spectra maxima at 358, 461, and 555 nm). Both the microscope and camera were connected to a computer system installed with image software (NIS Element BR-3.2; Nikon), which included image merging and a fluorescence intensity analyzer. For laser confocal microscopy, the same microscope was used and was equipped with a laser emission and detection system with three different channels. The optical scanning and image processing tasks were run by the Nikon EZ-C1 ver. 3.80 program (Nikon), and they included the reconstruction of Z-stack images into projections or 3D images.

**Table 1.** List of antibodies used.

Antibody	Mono/poly	Dilution	Host	Catalog #	Company
Laminin β2	monoclonal	1:100	Rat	#05-206	Millipore
Type IV col	polyclonal	1:10	Goat	AB769	Millipore
Type II col	monoclonal	1:100	Mouse	CP18	Millipore
Elastin	monoclonal	1:50	Mouse	MAB2503	Millipore
Tuj1	polyclonal	1:200	Rabbit	#04-1049	Millipore
Tuj1	monoclonal	1:200	Mouse	MAB1637	Millipore

## Results

**Figure 1.**
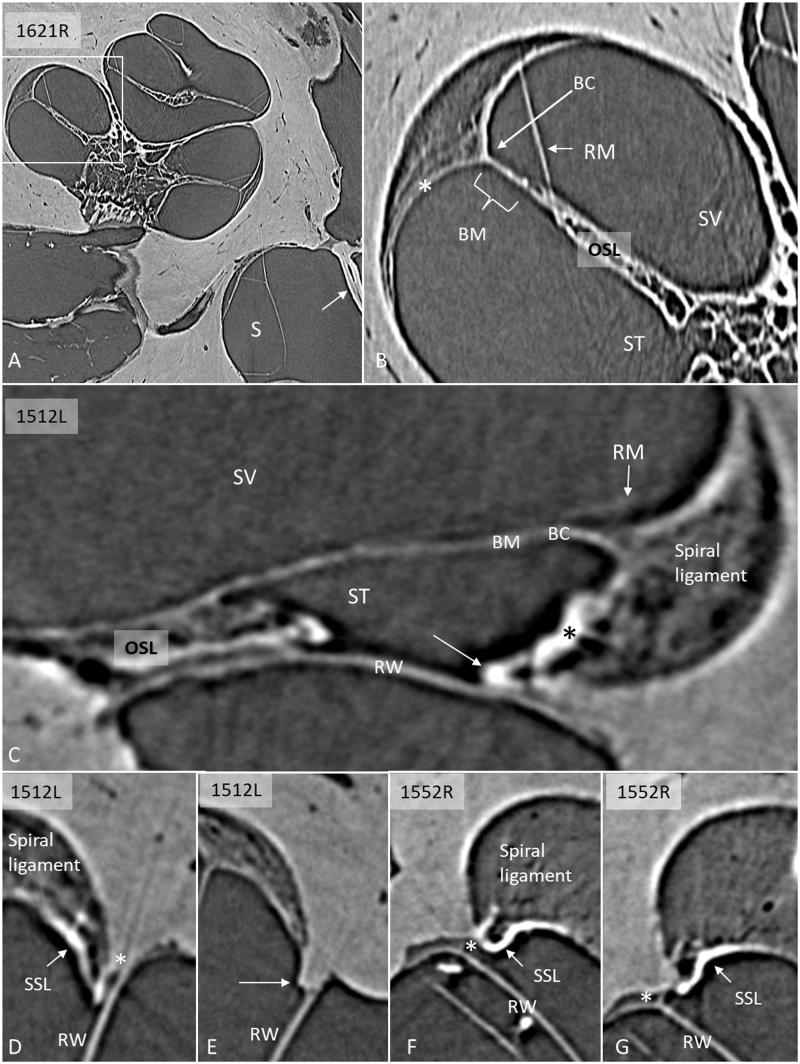
A: SR-PCI of a right human cochlea. Framed area is magnified in B. The saccule (S) and stapes plate (arrow) can be seen. B: Cochlear tissue is detectable, including Reissner’s membrane (RM), SL, and BM. C: SR-PCI of a left human ear at the level of the RW. The RM, BM, SL, and round window membrane (RWM) are clearly visible, as well as the limbus spirale. The SL facing the ST shows increased contrast (* and arrow). D–G: Sections showing the lateral attachment of the RW near the SL. There is some contrast enhancement of the ST wall facing the RW (arrows, SSL). There is often a space (*) between the RWM and the LW. (BC: basilar crest; BM: basilar membrane; LW: lateral wall; OC: otic capsule; OSL: osseous spiral lamina; RM: Reissner’s membrane; RW: round window; RWM: round window membrane; S: saccule; SL: spiral ligament; SR-PCI: synchrotron radiation phase contrast imaging; SSL: secondary spiral lamina; ST: scala tympani; SV: scala vestibuli).

**Figure 2.**
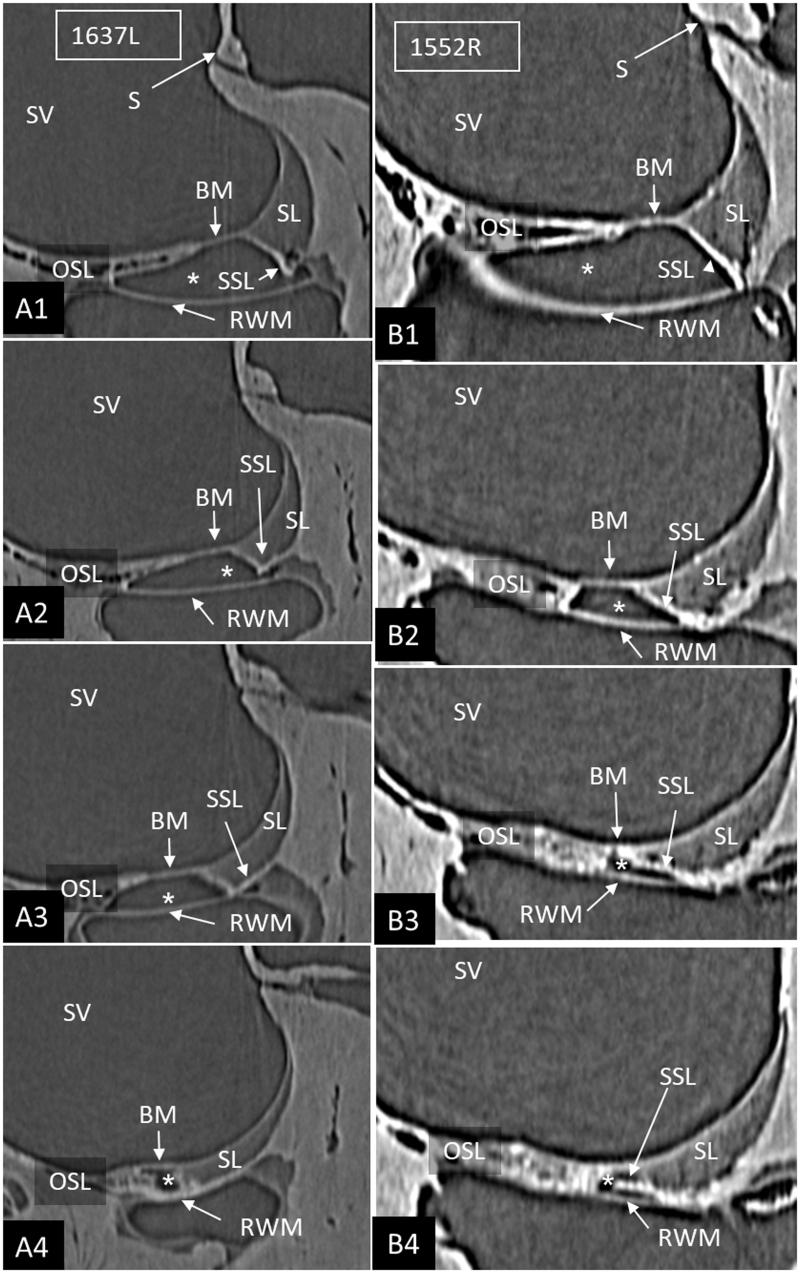
Serial SR-PCI sections from two right ears (A1–4; B1–4) at the cul-de-sac (*) of the ST space (*). The RWM extends basally beyond the level of the BM, and they do not seem to unite. The SSL separates the RWM from the BM (B3, 4) and the SL (A2–4). (For abbreviations, see legend to Figure 1).

SR-PCI reproduced the human cochlear soft tissues, such as the SL, the round window membrane (RWM), Reissner’s membrane (RM), and the BM (Figure 1). On radial sections, the BM was well-defined from the spiral limbus to the narrowing wedge of the BC at the lateral wall. The SL was well-delineated, and the part facing the scala tympani (ST) at the RWM often showed increased contrast (Figure 1(B)). These density areas varied among the bones and seemed to correspond macroscopically to the SSL. The relationship between the RWM and the BM was examined on serial SR-PCI sections in three specimens where the entire RWM was included (1637R, 1512R, and 1552R). The membrane extended beyond the basal end of the BM with no physical contact. A thin ledge of tissue was sometimes sandwiched between and separated the structures (Figure 2(B4)). The SSL also separated the RWM from the SL with no observed physical contact between them. Also, at this location, the primary osseous spiral lamina (OSL) merged with the SL. The increased contrast density of the ST wall faded as the distance between the RWM and the SL increased anteriorly into the cochlea.

### SR-PCI and 3D rendering

**Figure 3.**
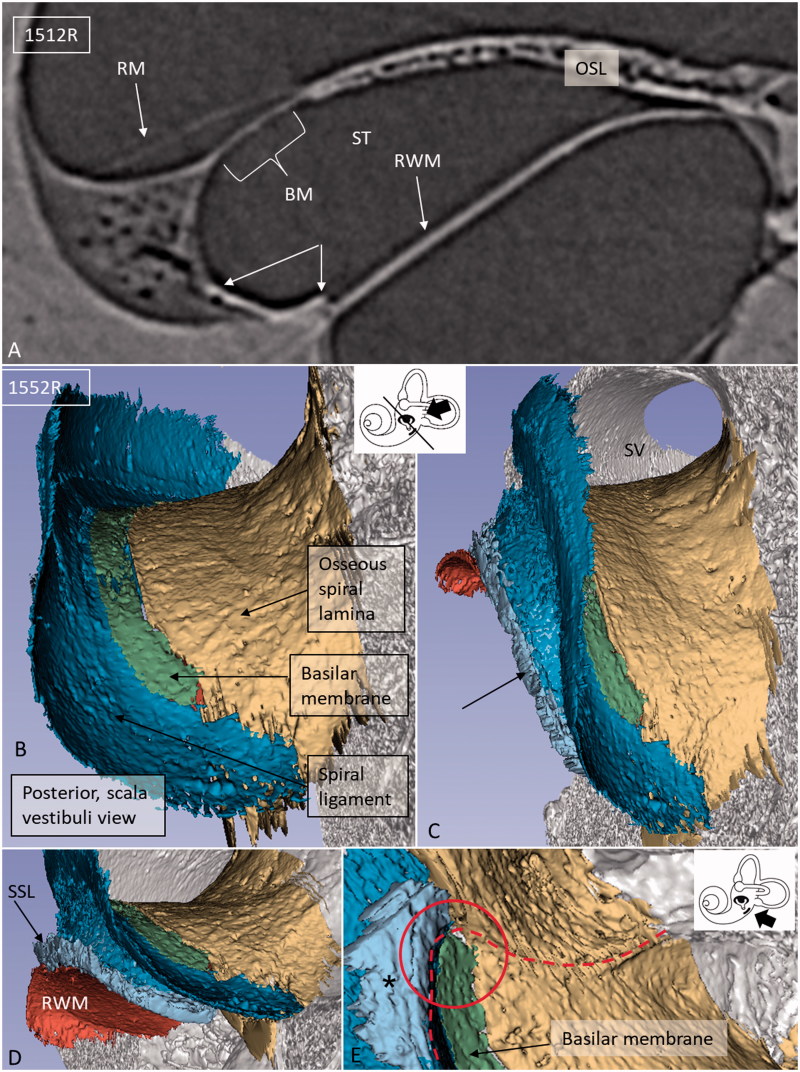
A: SR-PCI section of a left ear showing RM, SL (arrows), and RWM at the cul-de-sac of the endolymphatic space. There is an increased contrast (arrows) of the inferior region of the SL facing the ST. B: SV view of the 3D reconstructed tissues in the same cochlea. The basal end of the BM is seen together with the SL (blue) and OSL (yellow). C: Slightly angled view demonstrates the SSL (arrow). D: Postero-inferior view shows the BM in the SV and the external surface of the RWM with surrounding SSL. E: Infero-lateral view of the basal end of the BM where it joins with the SSL (*), SL, and OSL (encircled). (For abbreviations, see legend to Figure 1).

**Figure 4.**
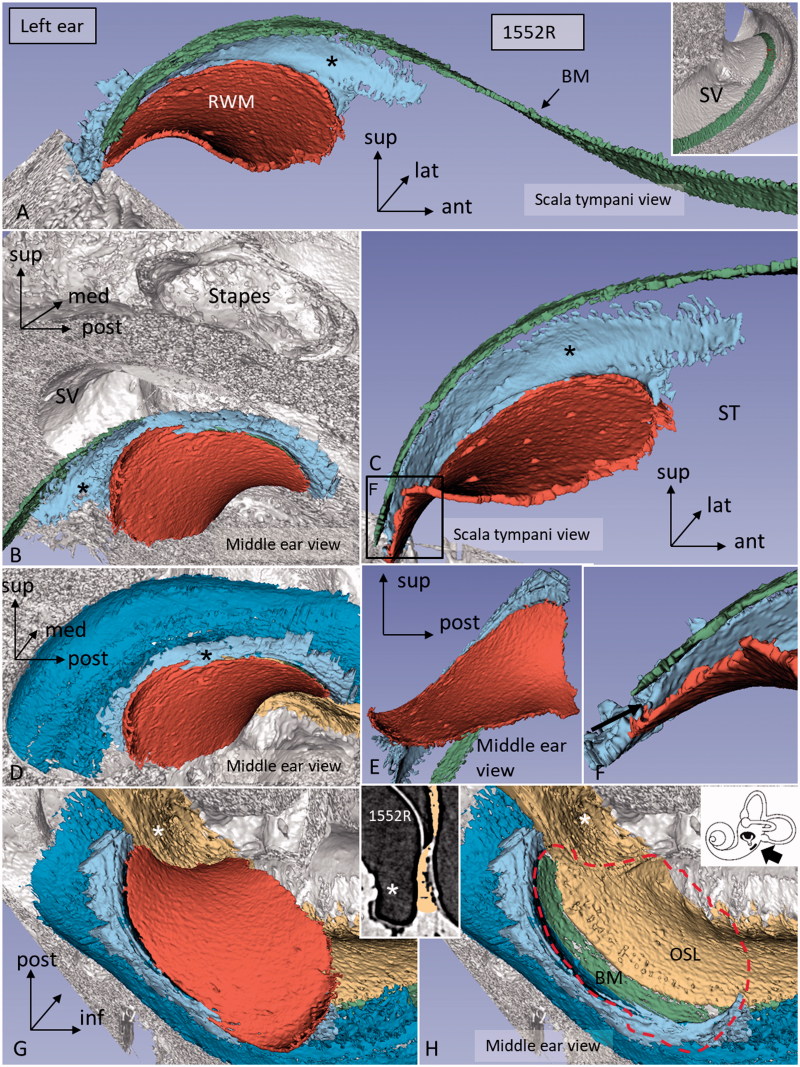
Different angular views of the 3D reconstructed RWM and neighboring soft tissues in a left human ear. A: Infero-medial view shows the relationship between the BM and the SSL (*). B: Same ear viewed from the middle ear displays the relationship between the RWM and stapes. The posterior portion of the RWM lies almost horizontal. C: Infero-medial view of the basal end of the BM and the RWM. Framed area is magnified in F (* = SSL). D: Lateral view of the SL (dark blue) (* = SSL). E: Infero-lateral view with conical shape. F: Magnified framed area in C. The BM is separated from the RWM (arrow). G: Infero-lateral view of the RWM and SL (blue). The close relationship between the SL and RWM is seen. H: Same view as G after removal of RWM (delineated). Inset shows a single SR-PCI section of the medial wall of the round window niche (RWN) (*) and the OSL (yellow). (For abbreviations, see legend to Figure 1).

**Figure 5.**
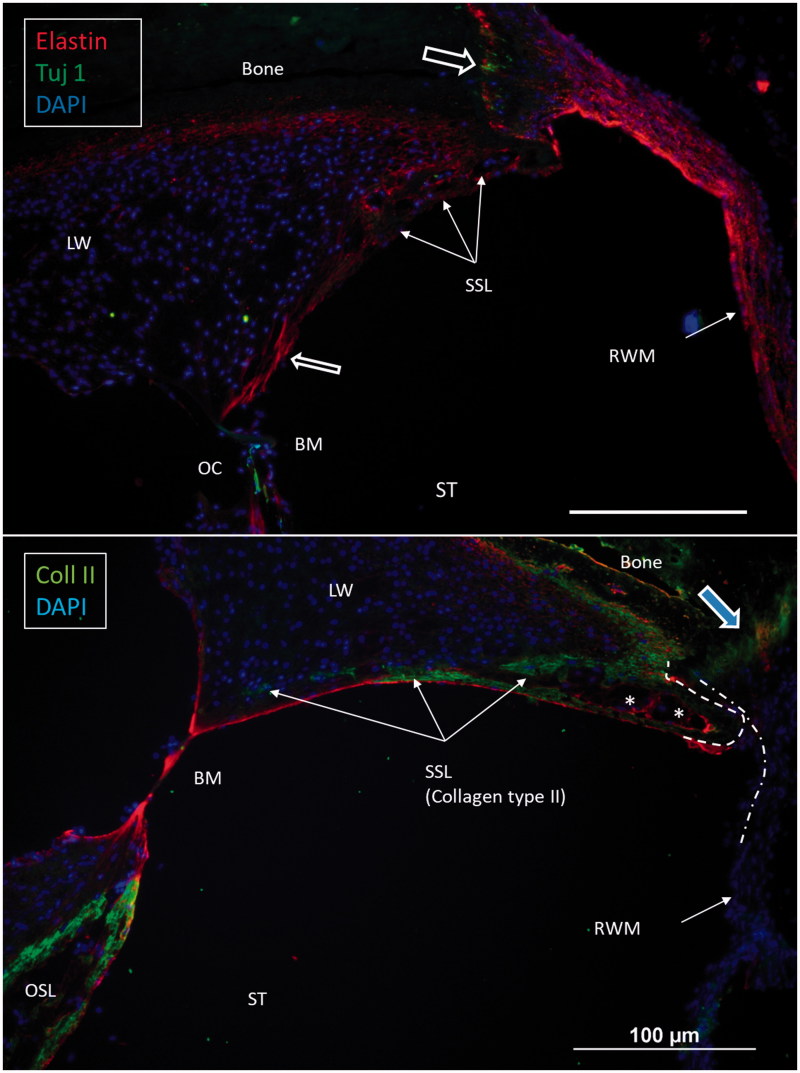
Confocal immunohistochemistry of the human cochlea at the level of the RW. Upper image: The RWM expresses elastin. Some elastic fibers radiate between the BM and the SSL. Lower image: The SSL expresses type II collagen. The OSL also expresses type II collagen. (For abbreviations, see legend to Figure 1).

The 3D Slicer software program reproduced the twisted ‘hook’ region, and the soft tissue increased the understanding of the complex anatomy. Color separation of anatomical structures on each section further enhanced the conception of the topographic anatomy (Figures 3–5). Furthermore, bones were made transparent, and cropping also improved visualization of structures otherwise disguised. The 3D anatomy of the entire RWM and the associated structures was characterized in three specimens, and 3D printing verified the complex shape. Results showed that the RWM was positioned in the same vertical plane as the oval window (Figure 4(B)). The membrane bulged both inwardly and outwardly in the horizontal and vertical planes, respectively. It angled antero-inferiorly, laterally, and horizontally. Also, it was ovoid with the longest diameter directed antero-posteriorly, and it was plough- and fan-shaped with two pointed ends (Figure 4(E)). One end was directed postero-inferiorly and one posteriorly. The postero-inferior end lay medially and was U-shaped (Figure 4(A,C)) and represented the point where the curved RWM approximated the OSL and attached to the bone near the opening of the cochlear aqueduct. The acoustic crest lay anteriorly to this point and formed a variably sized impression on the RWM rim. The posterior pointed end was the region where the RWM approximated the BM and SL. Between these points ran the basal portion of the OSL that sometimes faced the medial wall of the round window niche (RWN) (Figure 3(E); Figure 4(G,H)). The posterior curved shape of the RWM seemed to be generated by the hook rotation. This part was located almost horizontally (Figure 4(D)). Antero-superiorly, the RWM curved outwardly at the bony attachment.

Viewed from the scala vestibuli (SV), the 3D reconstructions showed that the lateral wall and the OSL merged to circumscribe the basal end of the BM and continued a short distance posteriorly and then faded (Figure 5(A,B)). Viewed from ST, the blind end of the BM could be realized relative to the LW (Figure 3(D,E); Figure 4(G,H)). The SSL reached the posterior level of the BM end and separated the BM from the RW. The SSL surrounded the postero-superior rim of the RWM and occupied the caudal part of the ligament wall facing the ST (Figure 4).

### Confocal IHC

Confocal microscopy showed that the caudal SL facing the ST at the RW often expressed type II collagen. The staining faded against the BC and BM. It was also prominent in the primary OSL, the inner surface of the otic capsule (OC), and the bony insertion of the RWM (Figure 5(A)). The RWM expressed elastin together with tissue located between the BC and the SSL. Tuj1-positive nerve fibers were found at the organ of Corti and the bony insertion of the RW (Figure 5(B)).

## Discussion

Mammals with low-frequency hearing have an SSL only in the basal turn, while mammals with high-frequency hearing seem to have a prominent SSL along the entire cochlear duct (1,15). In the horseshoe bat, the SSL was described as a substantial heart-shaped shelf of bone on the outer bony wall containing blood vessels. In radial sections, the tip points towards the BM, and, together with the enlarged SL, it may play a role for hydro-mechanical frequency analysis. The SSL is also prominent in rodents and guinea pigs where it seems to support the BM to the LW (1,16). In humans, the SSL appears to be limited to the lower part of the basal turn (3) or to a short region around the posterior and superior margins of the RW (4). In macerated bone specimens, its shape can be studied macroscopically to reach a short distance into the cochlea (4). This could also be verified in the present study. In recent investigations, we used high-resolution IHC (17,18), electron microscopy techniques (19), and micro-CT; the last-mentioned provided additional information about the 3D bony cochlear anatomy at the RW (20). An SSL was perceived to be more or less ossified. The OSL, BM, and LW could be seen to meet at one point. This point was named the ligament/lamina fusion point (LLFP) (20).

**Figure 6.**
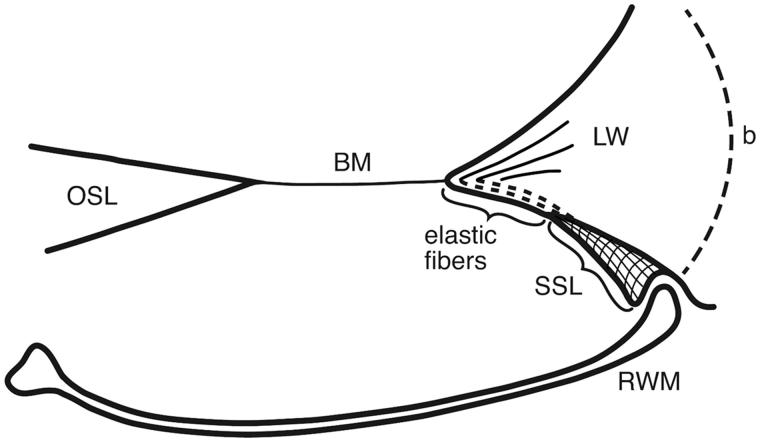
Drawing showing the principal arrangement of the BM and its attachment to the LW at the RWM in a human cochlea. The BM contains radial fibers which reach the BC and radiate into the SL. Fibers express the elastin path between the BC and the SSL. (b: bone; for other abbreviations, see legend to Figure 1).

Here, 3D SR-PCI reconstruction showed the blind end of the BM from both the SV and ST aspects in great detail (Figure 4(G,H)). The fissure between the primary OSL and the BC was bridged by an identifiable BM (Figures 1–3). SR-PCI even reproduced the RM, a 3-micron thick, two-cell-layer sheet separating the endolymph from the perilymph. It was seen at the blind end (cul-de-sac) of the endolymphatic space, while the fine reunion duct near the cochlea was difficult to perceive. The point that defines the commencement/cessation of the human BM was visualized. The SL wall facing the SV reached beyond the level of the BM where it fused with the OSL. An analogous arrangement was observed when viewed from the ST side (Figure 3(E)). Here, its relation to the RWM could also be established. Despite the proximity of the BM to the RWM, they never seemed to have physical contact with each other, and the SSL sometimes seemed to separate these structures. The results suggest that the SSL both mechanically supports the BM (that may be under some tension) and attaches it to the lateral wall. The elastin expression between the BC and SSL could serve to increase the compliance of the BM (Figure 6). The SSL may also shield the BM mechanically and acoustically from the impending RWM to avoid interfering motions. Another function could be that the SSL acts as a barrier and protects the LW from noxious agents reaching it from nearby infection-prone areas. IHC indicates that it is composed of type II collagen.

The RWM was initially described by Scarpa in 1772 (21). Its embryologic development was thoroughly analyzed by Anson in 1953 (22). It was initially used as a pathway for the insertion of electrodes in connection with cochlear implantation (23–27). This gateway was later abandoned due to the emergence of more laborious electrode arrays, but later gained new use, particularly in connection with hearing preservation surgery. In 1987, Franz et al. (28) studied the surgical anatomy of the human RWM in connection with cochlear implant (CI) surgery. They described its conical shape and a bony SSL in the basal part of the cochlea. The RWM consisted of an anterior vertical and a posterior horizontal part forming a right angle to each other. The conical shape of the RWM was also seen in the present investigation when viewed from the infero-lateral aspect (Figure 4).

Variations in the anatomy of the hook region are noticeable, as earlier shown by several authors (4,8,29–31). In a study by Li et al. (32), the anatomy of the RWM and the hook region was described with implications for CI and other endocochlear surgical procedures. They created a 3D model of the human cochlea from celloidin sections from a 14-year-old adolescent. Studies suggested that the width of the BM diminishes infinitesimally at the end of the cochlea. We found that the width was fairly constant along the hook region and was estimated to be around 0.2–0.25 mm without recognizable narrowing against the cochlear blind end. These observations may have surgical significance. Sound resolution depends on the stiffness and elasticity of the BM, which alters by a factor of 100 from base to apex in human cadaver ears (5). Electron microscopy showed that it is thicker in the base than in the apex and laterally, while, at the BC, it narrows and forms a wedge with a large number of radial filaments that spread out in the ligament (33). Thus, in humans, as well as in several other mammalian species, the thickness of the BM varies both longitudinally and radially (34,35), suggesting that not only the width of the BM is relevant for the mechanical frequency maps. The most conspicuous difference in humans compared to animals is the absence of a pars pectinata and arcuata in humans.

This microanatomy may be considered at CI surgery to preserve the structural integrity and avoid endolymph fistula, traumatization, and fibrotic reactions. The BM is fragile and easily perforated by CI electrodes, especially when inserted deep into the cochlea. Also noteworthy is the horizontal location of the dorsal RWM, which may be considered in the application of middle ear probes. The investigation clearly showed that there is a close proximity between the OSL and the medial wall of the RWN where high-frequency nerve fibers are lodged (Figure 3(E); Figure 4(H)). These neurons may be directly reached from the middle ear. Our SR-PCI investigation shows that the human cochlea is also endowed with a SSL but restricted to the RW area. It may act to suspend the BM but could also play a physiological role at the filtering of high-frequency sounds in the hook area of the human cochlea. This anatomy should be considered at cochlear implantation aiming at hearing and structural preservation.
